# Vaccinating the Vulnerable: How Do We Protect Those Experiencing Homelessness?

**DOI:** 10.1177/21501327211041211

**Published:** 2021-08-24

**Authors:** Lucy Jane McCann

**Affiliations:** 1 University of Leeds, West Yorkshire, England

Dear Editor,

I read with great interest the commentary by Katzman et Katzman which highlights the challenges of the COVID-19 vaccine hesitancy and explores possible strategies to tackle this challenge.^1^ As a final year medical student who delivers vaccination counseling at an inner-city general practice in the UK, I have experienced first-hand the difficulties described. However, our patient population poses unique and complex challenges to vaccination rollout—it comprises entirely of socially excluded groups such as those experiencing homelessness. Whilst the UK vaccine program has progressed expeditiously to vaccinate 88% of those eligible with a first vaccine, our general practice falls substantially behind; only 47% of our patients are vaccinated to-date.^2^ These figures are particularly concerning as those experiencing homelessness are high-risk regarding COVID-19. I would like to draw from the anecdotal but extensive experiences we have faced vaccinating this vulnerable group to build on Katzman and Katzman’s commentary suggestions and contribute to the dearth of literature on this topic.

In general, we have found 3 predominant barriers to vaccinating those experiencing homelessness. Firstly, and arguably the most significant, is vaccine convenience. The unpredictability of our patients’ lives has considerably affected vaccine logistics. For people experiencing homelessness, there are a myriad of competing priorities which often override the desire to get vaccinated, such as acquiring food or shelter. To maximize vaccine convenience and eliminate any additional time or money needed to attend the practice itself, we have been using a mobile clinic to provide vaccines for patients at hostels or whilst on outreach visits. Another method is to reach patients when they attend for an appointment unrelated to vaccination; we check if the patient has been vaccinated, and if not, offer an immediate appointment at our vaccine clinic operating within the practice. These steps have eased the process for many of our patients by improving accessibility of services and hence promoted uptake.

Secondly, vaccine confidence is also lacking within this group. We have found that some patients mistrust healthcare workers due to previous discriminatory experiences, leading to them lacking confidence in services provided. Although few studies to date review attitudes specifically towards COVID-19 vaccines within this group, 1 recent study from France found that those living in homeless shelters had elevated levels of vaccine hesitancy.^3^ This challenge can be overcome by applying the same evidence-based tools described by Katzman and Katzman, such as using a patient-centered approach and peer to peer counseling. Such techniques have proven beneficial within our practice. However, clinicians must adapt how they disseminate information to better target this disadvantaged group; patients respond well to concise, clear, and non-judgmental discussions. The longitudinal nature of primary care offers an opportunity for clinicians to build relationships with patients and foster trust which will help reverse their negative perceptions of healthcare. Until then, a tailored approach to vaccine counseling training may benefit practices whose patient populations encompass those experiencing homelessness.

Finally, there is an immense challenge in providing 2 vaccinations at the recommended time intervals. This can be because patients attend for second vaccinations when they have got their first vaccine elsewhere. If the vaccination has not been formally recorded, this can be a challenge as patients are often unsure what vaccine they have had, or when. Similarly, we sometimes have difficulties contacting patients for a second vaccine as their telephone numbers have changed or they have moved addresses due to unstable accommodation. Other patients have thought that 1 vaccination was enough to protect them from COVID-19 and consequently not attended second appointments. Single-dose vaccinations are an ideal solution to this problem, such as Johnson and Johnson’s. As this is not yet readily available in the UK, we have taken several alternative steps to boost second vaccination rates. At each appointment, we check contact details are up to date and document any vaccinations given clearly within patient notes. We also provide patients with both written and verbal information on the following to empower them to have the best opportunity to get a second dose incase their circumstances change:

1. advice on why a second vaccination is necessary
2. which vaccine they have had
3. where they can get a second dose
4. when they will need a second dose

Once single-dose vaccinations are available, they should be prioritized for patients with less predictable lifestyles, such as those experiencing homelessness.

Vaccinating the vulnerable is essential for global management of COVID-19 and to minimize socioeconomic inequalities. General practices should adjust their vaccine rollout to ensure they provide equitable vaccine coverage and do not overlook those experiencing homelessness.
